# Local structure controls the nonaffine shear and bulk moduli of disordered solids

**DOI:** 10.1038/srep18724

**Published:** 2016-01-06

**Authors:** M. Schlegel, J. Brujic, E. M. Terentjev, A. Zaccone

**Affiliations:** 1 University of Cambridge, Department of Engineering, Trumpington Street Cambridge CB2 1PZ, UK; 2 Cavendish Laboratory, University of Cambridge, JJ Thomson Avenue CB30HE Cambridge, UK; 3 Physics Department, New York University, New York, NY 10003, USA; 4 Department of Chemical Engineering and Biotechnology, University of Cambridge, New Museums Site, Pembroke Street, CB2 3RA Cambridge, UK

## Abstract

Paradigmatic model systems, which are used to study the mechanical response of matter, are random networks of point-atoms, random sphere packings, or simple crystal lattices; all of these models assume central-force interactions between particles/atoms. Each of these models differs in the spatial arrangement and the correlations among particles. In turn, this is reflected in the widely different behaviours of the shear (*G*) and compression (*K*) elastic moduli. The relation between the macroscopic elasticity as encoded in *G*, *K* and their ratio, and the microscopic lattice structure/order, is not understood. We provide a quantitative analytical connection between the local orientational order and the elasticity in model amorphous solids with different internal microstructure, focusing on the two opposite limits of packings (strong excluded-volume) and networks (no excluded-volume). The theory predicts that, in packings, the local orientational order due to excluded-volume causes less nonaffinity (less softness or larger stiffness) under compression than under shear. This leads to lower values of *G*/*K*, a well-documented phenomenon which was lacking a microscopic explanation. The theory also provides an excellent one-parameter description of the elasticity of compressed emulsions in comparison with experimental data over a broad range of packing fractions.

One of the overarching goals of solid state physics is to find a universal relationships between the lattice structure of matter in the solid state and its mechanical response. From this point of view, it is important to simplify the details of the interactions between the building blocks (atoms, particles) in order to single out the relevant physics and general laws. The framework of lattice dynamics successfully provided the link between atomic-level structure and macroscopic properties of simple crystal lattices^1^. Our understanding is instead much more limited when structural disorder plays an important role, such as in glasses, liquids and other disordered states of matter^2^,^3^.

With the advent of computer simulations, it became clear that disordered solids, which are of paramount importance in many areas of technology and life sciences, cannot be described simply as perturbations about the crystalline order. In this context, an unsolved problem is the striking difference in the elastic deformation behaviour of random networks and random packings. For networks, the shear modulus *G* and the compression modulus *K* display the same dependence on the coordination number *z* which represents the average number of elastic springs per node of the network. Therefore, 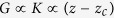, and both moduli vanish at the same critical coordination 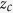 which is dictated by isostaticity. It is different for random packings where only the shear modulus scales *linearly* as 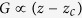, whereas the bulk modulus vanishes only at a coordination much lower than 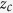. This means that packings have a comparatively larger bulk modulus, with respect to random networks, and remain well stable against compression also near, at, and even below the critical coordination where shear rigidity vanishes. This state of affairs has been revealed in simulation studies^2^,^4^, at least since the 1970’s^5^. Furthermore, the same phenomenon is well documented also in disordered atomic solids^6^ and non-centrosymmetric crystals (e.g. piezoelectrics)^7^.

However, there is no mechanistic understanding of this phenomenon, nor analytical theories able to describe it, beyond the somewhat obvious observation that the internal structure of packings is different from that of random networks, due to the self-organization and mutual excluded-volume of particles in the packing, which are absent in random isotropic networks. Below we provide a quantitative connection between structure and elasticity based on nonaffine lattice dynamics which shows that the local self-organization of the particles with excluded-volume leads to a higher degree of bond-orientational order^8^,^9^ in randomly packed structures compared to isotropic random networks. In turn, this leads to a significantly higher bulk modulus and a lower nonaffinity under compression.

## Results

### Nonaffine lattice dynamics

Our main tool is the Born-Huang free energy expansion^10^, suitably modified to account for the structural disorder in terms of the nonaffinity of the displacements (as explained below). In order to make analytical calculations, we neglect the effect of thermal fluctuations (i.e. we operate in the athermal limit, which is applicable to granular solids and non-Brownian emulsions), and we focus on harmonic central-force interactions between the particles. Thus we neglect both the bending resistance when the particles slide past each other, as well as the effect of stressed bonds. It is important to emphasize that both these effects can provide rigidity to certain lattices, which are otherwise floppy or unstable when only central forces between atoms are active. This fact is well known e.g. in the context of inorganic network glasses^10^,^11^.

The key to understanding the elasticity of amorphous lattices is nonaffinity^10^. In a nutshell: the applied external deformation induces a deformation at the microscopic level of interatomic bonds. If the interatomic displacements are simply proportional to the applied overall deformation field, then the deformation is called *affine*, and one can expand the free energy in powers of small interatomic displacements and take the continuum limit of the microscopic deformation for either shear deformation or compression^1^. In other words, the microscopic interparticle displacements are directly proportional to, and uniquely determined by the applied macroscopic strain. Differentiating the free energy twice with respect to the macroscopic strain yields the shear modulus *G* and the bulk modulus *K*, depending on the geometry of the applied deformation (shear or hydrostatic compression, respectively).

**Figure 1:**
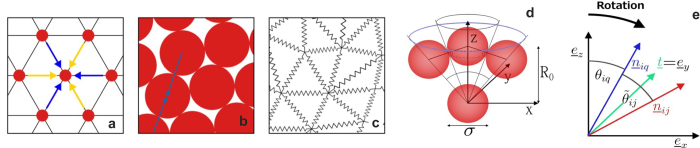
Geometry of particles, bonds and forces. (**a**) In a centrosymmetric lattice the forces acting on every particle cancel by symmetry and leave the particle force-free. Hence no additional displacements are required to keep local mechanical equilibrium on top of the affine displacements dictated by the applied strain. (**b**) In a jammed packing, there is a remarkable degree of local orientational order: due to excluded-volume correlations it can still happen that two particles make an angle equal to 180° across the common neighbour at the center of the frame, leading to cancellation of local forces. This effect is significant under compression, thanks to isotropy, but negligible under shear. (**c**) In a random network, the probability of having this cancellation of forces is much smaller. In this case, nonaffine displacements are required on all particles (nodes) to keep local equilibrium under the non-vanishing sum of nearest-neighbour forces. This limit has the strongest nonaffinity and the lowest values of elastic moduli. (**d**) The excluded volume cone: a bond, for example along the *z*-axis, leads to an excluded-cone where no third particle can exist. 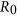 is the equilibrium bond distance, *σ* represents the diameter of the particles. (**e**) The frame-rotation trick to evaluate the contributions of local excluded-volume correlations to the nonaffine elastic moduli. Here, for simplicity, only the special case of 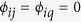, i.e. both *ij* and *iq* lying in the plane *xz*, has been illustrated.

As was first realized by Lord Kelvin^12^, and more recently emphasized by Alexander^10^ and Lemaitre and Maloney^13^, the affine approximation is strictly valid only for centrosymmetric crystal lattices. The reason becomes evident if one considers the forces which are transmitted to a test atom in the lattice upon deforming the solid. Every neighbour transmits a force which is cancelled by the *local* inversion symmetry in the centrosymmetric Bravais cell (see Fig. 1a below). As a result, there is no local net force acting on the atoms of the lattice in their affine positions, and the old affine free energy expansion^1^ suffices to correctly describe the elastic deformation. With a disordered or non-centrosymmetric lattice, the situation is different. The forces that every atom receives from its neighbours no longer cancel, because the local inversion symmetry is violated. The net force acting on every atom has to be relaxed via additional atomic displacements, called *nonaffine* displacements^13^. These motions, under the action of the disorder-induced local forces, are associated with a total work, which is an internal work done by the system (hence negative, by thermodynamic convention).

The work done by nonaffine displacements represents a quote of internal lattice energy which cannot be employed to react to the applied deformation. Therefore, the free energy of deformation can be written as 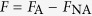, to distinguish the affine contribution 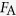 from the nonaffine contribution due to disorder^14^,^15^, 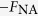. The fact that non-centrosymmetric lattices (e.g. piezoelectric crystals) are affected by nonaffine distortions of the primitive cell^16^,^17^, however, does not necessarily mean that they are unstable or soft. These materials are, of course, fully rigid and do exhibit a large value of shear modulus, provided that they have a sufficient atomic coordination, well above the isostatic limit, and a fairly large value of spring constants.

### Theory of elastic moduli

Upon carrying out the formal treatment with the standard dynamical (Hessian) matrix^18^ 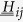 and the expression for the disorder-induced force (defined for the example of shear deformation *γ* in the 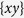 plane as 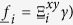, the nonaffine contribution to the free energy of deformation can be evaluated as shown in several places in the recent literature^13^,^14^. It has been shown that the elastic constants are given by 
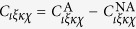
 with the nonaffine correction due to disorder given as


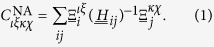


The affine part of the elastic constants is provided by the affine Born-Huang lattice dynamics, which is exact for centrosymmetric lattices: 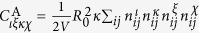. Here 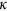 is the effective spring constant of the interatomic (interparticle) interaction, which is harmonic near the equilibrium, *V* is the total volume of the system, and 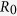 is the equilibrium separation length between nearest neighbours spheres of diameter *σ*. 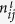 is the 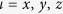 Cartesian coordinate of the unit vector which defines the orientation of the bond between two bonded neighbours *i* and *j*. In the nonaffine relaxation term, the force per unit strain acting on every atom is given analytically, for the case of shear deformation, by^13^ 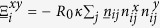. It is easy to check that 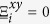 for a centrosymmetric lattices. As shown in ref. 14, under the assumption of central-force interaction, and for a random network of equal harmonic springs with number density of nodes 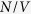, the shear modulus can be evaluated analytically as


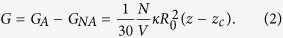


The proportionality to *z* is contributed by the affine term 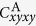 above, where the sum 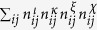 can be evaluated in mean-field averaging, 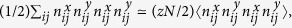
where the quantity 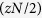 represents the total number of bonds in the system. The factor 1/2 in front of the 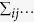 is required because the sum counts the bonds twice. Further, 
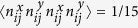
 for a random isotropic distribution of bond orientations.

The nonaffinity of the amorphous solid is encoded in the quantity 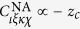, which defines the critical number 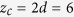 of bonds at which the shear modulus vanishes by virtue of the non-affine softening mechanism.

This result is valid for random networks where bonds have randomly distributed orientations in the solid angle. In that model, any bond-orientational order parameter is identically zero and the average rotational symmetry is isotropic. For the more general case where correlations between bond-orientation vectors of nearest-neighbours are important, it can be shown (see the Supplementary Information) that the nonaffine correction term reduces to the following form, after replacing the sum over bonds by the average:


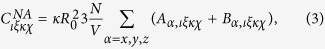


where 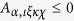 and 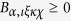 are defined as follows:


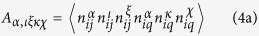



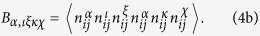


Here 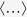 represents an angular average, in the solid angle, over the orientations of bonds *ij* and *iq* as explained in^15^. It is important to note that in 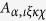, we average over all possible orientations of two bonds to the atoms *j* and *q*, respectively, measured from a common atom *i*. For the average in 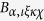, one only needs to consider bonds between the particles *i* and *j* as discussed in^14^. Hence, it is evident that 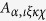 is non-zero only if the orientations of the two bonds *ij* and *iq* are correlated (that is, the orientation of *ij* does depend on the orientation of *iq*, and vice versa). If there is no correlation, meaning that given a certain orientation of *iq* in the solid angle, *ij* can have any random orientation in the solid angle with the same probability, then 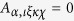. This is so because the average can be factored out into the product of two averages of triplets each of the type 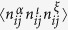, and each angular average vanishes separately, as one can verify by insertion.

The limit where any two bonds *ij* and *iq* are uncorrelated, and 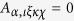, defines the geometry of the random network^4^ (Fig. 1c). The random network limit represents the case where nonaffinity makes the largest negative correction to the elastic constants, thus softening the material. The random network is thus the opposite extreme to the perfect centrosymmetric hard crystal.

In the random network model, which served for long time as a structural model for many inorganic glasses^11^,^19^, the nodes are just point-atoms with zero volume, 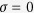. This is a very important feature because the absence of any excluded-volume hindrance between such atoms allows them to be placed at random positions in space. Such a model is clearly applicable only to systems where the bond length is much larger than the atomic diameter *σ* (which is the case for network glasses and some amorphous semiconductors). The limit 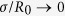 thus corresponds to the random network model. The opposite limit, 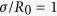, corresponds to the jammed packing, where spherical particles are barely touching their neighbours. In this limit, the excluded-volume repulsion between spheres in close contact plays a very important role in the self-organization and in the local structure of the packing. In particular, due to excluded-volume, there are restrictions on the available portion of solid angle where a nearest-neighbour can sit. It is therefore significantly more likely, in comparison with the random network case, that a particle *j* makes an angle of 180° with a particle *q* directly across a third particle *i* placed at the center of the frame (Fig. 1b), due to the existence of sectors in the solid angle (as measured from the central particle) that are forbidden. Hence, the local orientational order in the jammed packing, well documented in previous structural studies^8^,^9^, is important also in the determination of elastic moduli. In the following we are going to focus our detailed calculations on the jammed packing limit with 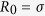.

We implemented a minimal model, inspired by the granocentric model of granular packings^20^, for the excluded-volume correlations which allows an explicit evaluation of the two-bond angular-correlation terms 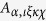 for jammed packings. If the bond *iq* has a given orientation in the solid angle, parameterised by the pair of angles 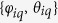 then, clearly, the bond *ij* can have any orientation in the solid angle apart from those orientations delimited by the excluded cone depicted in Fig. 1d. The angular average for the orientation of *ij* is thus restricted to the total solid angle Ω minus the excluded cone, which gives the allowed solid angle as 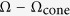, with 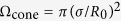. The probability density distribution *ρ* of bond orientations is taken to be isotropic for *iq*, that is 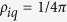. For *ij*, instead, the probability that it takes a certain orientation is a conditional one, because it depends on the orientation of *iq*. Hence, the conditional probability for the orientation of *ij* is 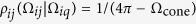, for 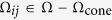, and 
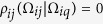
 for 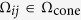. In the section below we use these considerations to evaluate the excluded-volume correction to the nonaffine moduli encoded in 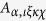.

### Evaluation of the excluded-volume correlations term in the moduli

The excluded-volume correlation term contributing to the elastic moduli is given by


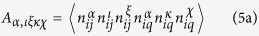



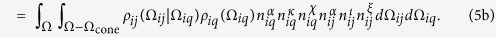


To evaluate the above integral it is necessary to first identify the correlation between *ij* and *iq* and then devise a strategy to evaluate the integral in the above equation.

A solution can be found by exploiting the symmetry of the problem, and, in particular, the rotational invariance. The local Cartesian frame centered on the particle *i* is rotated such that the *z*-axis (from which the azimuthal angles 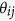 and 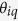 are measured) is brought to coincide with the unit vector 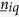 defining the orientation of the bond 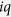 (see Fig. 1e for illustration of the special case where *iq* and *ij* lie in the *xz* plane). This trick reduces the number of variables in the problem: instead of dealing with two sets of angles, 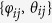 and 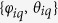, we need to consider only one set 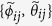, which gives the orientation of the bond *ij* in the rotated frame. Upon suitably defining the rotation matrix, the above integral is much simplified.

The rotation is defined around an axis 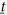 (parallel to 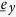 in the special case of 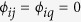 illustrated in Fig. 1e), and perpendicular to both 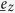 and 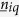, with an angle of 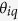 (usual convention of rotation: counter clockwise if axis vector points in the direction of the viewer). Here, 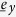 and 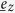 denote the unit vectors along the *y* and *z* axis, respectively, of the Cartesian frame centered on particle *i*. Therefore, the unit vector t defining the rotation axis is:


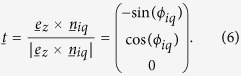


The rotation matrix 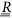 is defined by the Rodrigues’ formula^21^


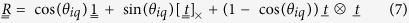


where 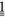 represents the identity matrix. Further, we defined


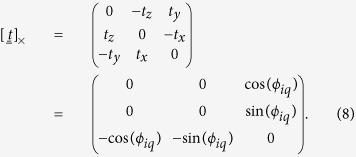


Next, we look at the integral 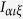 defined as:


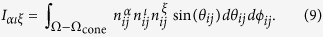


This integral occurs in the expression for 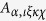, and considering that 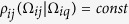 in the allowed solid angle 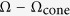 for *ij*, we have factored 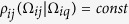 out of the *ij* integral leaving a product between 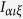 and 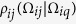 inside the integral of Eq. 5(b),


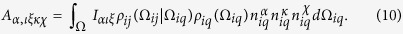


As is shown in the SI, in the new rotated frame, one obtains:


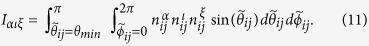



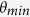
 is determined by the excluded volume cone as 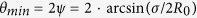.

We recall that 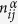 is defined as the *α* Cartesian coordinate of the bond unit vector 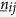 and is related to the bond unit vector of the rotated frame 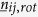 via 
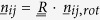
, with 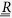 given by Eq. (7). The bond unit vector in the rotate frame 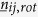 is defined by the pair of angles 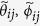 which represent the integration variables in Eq. (11). Therefore, we can now use Eq. (11) together with Eq. (10) to arrive at the following expression for 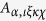:


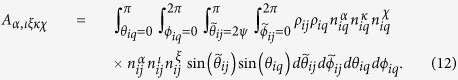


With the last Eq. (12), we have reduced the original integral for 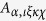 to a much simpler integral with well-defined integration limits in the solid angle. The integral can be easily evaluated using 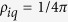, which


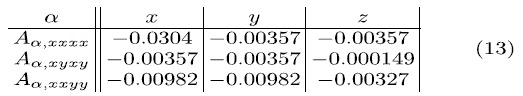


accounts for the fact that the orientation of *iq* can be freely chosen, whereas 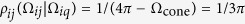 due to the restriction imposed by excluded-volume.

From the evaluation of the integral we obtain the following numerical values of the coefficients,

We also recall that 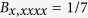, 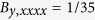, 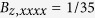 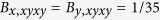, 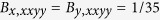, 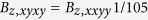 as obtained in ref. 14. Using these values of coefficients in Eq. (3), for shear in the *xy* plane we find: 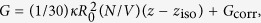 where 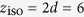 and the correction term due to excluded-volume correlations is 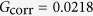, in units of 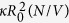. The anisotropy of the shear field leaves a small projection of the interparticle forces in the direction of the opposing bonds, which leaves nonaffinity nearly intact under shear.

## Discussion

The non-zero, though small, 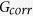 predicted by the analytical theory might be due to model approximations which are intrinsically different from approximations and assumptions done in numerical simulations. For example, we always overestimate the excluded-volume cone by not considering the deformability of the soft particles in jammed packings. If this was properly taken into account, it would lead to a smaller excluded-volume cone and weaker correlations, hence to a higher nonaffinity than predicted in this approximation. In turn, that would yield an even smaller, practically negligible, value of 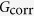. Another, though related, source of inaccuracy is the neglect of deviations from the average nearest-neighbour distance *R*_0_. These deviations are possible if the particles are allowed to deform slightly at contact. There are also other differences in terms of boundary conditions and the structure of the packing cannot obviously be exactly the same for theory and simulations. Further, we do not take into account *local* chemistry-related effects at the interface between grains/drops (which may control how the creation of excess contacts 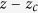 depends upon 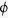 under different physico-chemical conditions^22^,^23^,^24^). This is so because we want to focus on the more general many-body physics which controls the mechanical deformation behaviour (i.e. how *G* and *K* vary with *z*).

In a similar way, for the bulk modulus we obtain 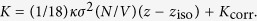
In this case 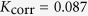, always in units of 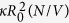, is significantly larger. The reason why 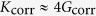 lies in the fact that the forces transmitted by neighbours are on average cancelling each other effectively under isotropic compression, though not to the same extent in shear. The latter is strongly anisotropic and causes the forces transmitted by neighbours to be misaligned such that the cancellation of nearest-neighbour forces with same orientation and opposite direction is not as effective. Our theoretical predictions match the known effect of vanishing of the ratio *G*/*K* at the rigidity transition^4^ 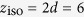. The analysis for the centrosymmetric crystal based on the affine assumption can be found in Born’s work and gives the constant ratio^1^ 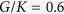, independent of *z*. The same ratio is also found in the simulations of ref. 2. This limit is captured by our general framework of disordered lattice dynamics, as both sets of coefficients 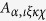 and 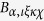 are identically zero for centrosymmetric crystals, giving 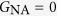 and 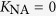.

We have seen above that the shear modulus does not completely vanish at the isostatic transition, but remains small and equal to 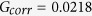, and that the ratio 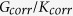 is about 0.26. Hence, our theory gives an order of magnitude 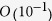, instead of 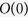, as many numerical simulations seem to suggest upon extrapolation to 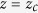. On the other hand, however, our theory is the only analytical approach which predicts a substantial difference, close to one order of magnitude, between *K* and *G*. In many amorphous and other non-centrosymmetric materials, the difference between shear and bulk modulus is about a factor 4, like for example in crystalline ice and quartz^7^,^25^, which is very consistent with our result.

**Figure 2:**
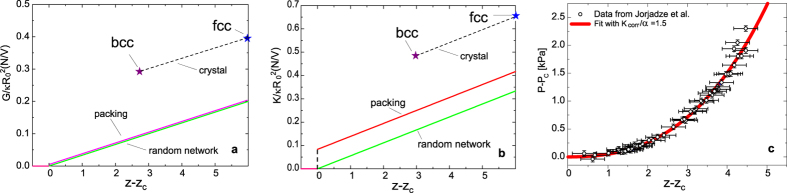
Theoretical predictions in different limits across the disorder spectrum. (**a**) Theoretical predictions for the shear modulus *G* near the isostatic limit 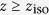, for crystals, jammed packings and random networks. The small term 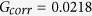 which contributes to the packing shear modulus has been neglected in line with the considerations presented in the text. (**b**) Similar predictions for the bulk modulus *K* for crystals, jammed packings and random networks, where now 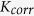 is making an important contribution to the packing bulk modulus. (**c**) Fit of experimental data of ref. 26 on compressed emulsion, using our Eq. (14) with the only fitting parameter given by 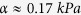.

Our theoretical predictions are presented in Fig. 2a,b for the shear and the bulk moduli, respectively. It is evident that the random network is the overall softest system because even if the shear modulus is basically the same as for the jammed packing (apart from the relatively small term 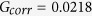 in the packing modulus which we neglected in the plot), its bulk modulus is significantly smaller. The reason is that the bulk modulus of the packing behaves closer to the affine deformation limit due to the reduction of nonaffinity caused by excluded-volume correlations, as explained above. Intriguingly, the same behaviour (soft shear modulus, quasi-affine bulk modulus) is well known to occur in atomic amorphous materials, such as amorphous Gallium^6^. In the random network, instead, the nonaffinity is strongest because no cancellation of forces due to local particle correlations can occur. This microscopic mechanism thus explains what observed in recent numerical simulations where this difference between packings and networks was investigated numerically^4^. What was interpreted as an “anomalous” behaviour, can be explained mechanistically based on nonaffinity.

Finally, our microscopic theory provides a quantitative prediction of moduli and of the discontinuous jump of the bulk modulus at the jamming transition, quantified by 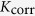. We introduce the shorthand 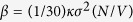 and 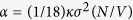 for the prefactors of *G* and *K*, respectively, for convenience of notation. Recalling that 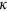 has units of 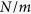, *σ* is a length and 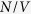 is in units of *m*^−3^, it is clear that *α* and *β* are measured in units of 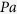, although here we discuss their calculated values in units of 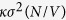. Calculating the slope 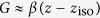, we find 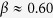 for the shear modulus, in good agreement with the value 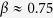 found in the simulations of Goodrich *et al.*^2^. For the jump in the bulk modulus at jamming, using the short-hand 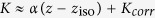, our theory gives 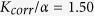, which is of the right order of magnitude but smaller than the value 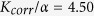 given by Goodrich *et al.*^2^. This discrepancy might be due to the obviously different approximations and assumptions done in numerical simulation protocols, which were discussed at the beginning of this section.

### Comparison with compressed emulsions

We also compared our prediction for the jump of compressibility with recent experiments on compressed emulsions^26^. In the experiment, different values of pressure applied to the packing were recorded, and the values of *z* corresponding to the different pressure values were measured using a fluorescent dye in the interparticle contacts between emulsion droplets. The output of this measurement is a curve relating 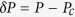 to 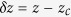, where we have to interpret 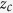 as the limit of isostaticity. The bulk modulus is defined in terms of pressure and coordination *z* via 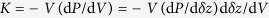. There is a one-to-one mapping between the volume fraction occupied by the drops, 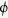, and the contact number, *z*, in compressed emulsions, which was determined empirically in ref. 26 to be 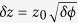, with 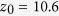, for their system. Using this relation, and the definition of volume fraction 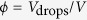, one obtains: 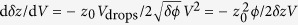. Upon replacing in the formula for *K*, we finally have a relationship between *K*, 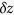, and 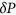, given by 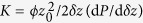. We can thus replace our theoretical expression for 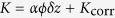 where *α* is the only fitting parameter containing the spring constant, and integrate the differential equation to get


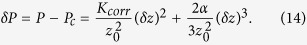


The one-parameter fit comparison between the analytical theory, given by Eq. (14) and the experimental data of ref. 26 is shown in Fig. 2c. The only fitting parameter is 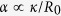 which is directly proportional to the spring constant of the drop-drop interaction, hence contains the dependence on the particular chemistry of the emulsion, and inversely proportional to the drop diameter. Our fitting accounts for both creation of excess contacts with pressure, and nonaffine particle rearrangements, and is able to provide a one-parameter fit of the data. In ref. 26 the same data were modelled by accounting for the creation of excess contacts only, and neglecting rearrangements, which requires two adjustable parameters. Hence, a more quantitative description of experimental data can be achieved using the new framework proposed here.

## Conclusions

We showed that the mechanical response of solids is strongly affected by the degree of local orientational order of the lattice, whether fully enforced (as in centro-symmetric crystals), low (as in random networks), or intermediate due to excluded-volume constraints in jammed packings). In particular, intermediate degrees of orientational order are very relevant for amorphous solids as documented by numerical simulations and experiments (see e.g. refs 8,9). Our theory shows that the lower the local orientational order, the stronger is the role of internal nonaffine deformations which always soften the mechanical response. With excluded-volume correlations, as in packings, there is significant local orientational order^9^ and two bonds can have the same orientation across a common neighbour, due to excluded-volume correlations. The forces transmitted by these nearest-neighbours cancel each other completely under compression, thus considerably reducing nonaffinity and softening for the compression mode. For lattices with strong excluded-volume like random packings (but also atomic materials like amorphous Gallium), our theory predicts that the bulk modulus can be a factor of 4 larger than the shear modulus, which is in semi-quantitative or at least qualitative agreement with both simulations^2^,^4^ and experiments on atomic^6^ and molecular materials^7^. Furthermore, our theory provides an excellent quantitative description of the dependence of the bulk modulus of compressed emulsions on the microscopic coordination number, with just one fitting parameter in the comparison with experiments^26^. We also expect that our lattice dynamics framework for materials that lack inversion symmetry can lead to a better understanding of the role of phonon lattice instabilities on the critical temperature of superconductors^27^.

## Additional Information

**How to cite this article**: Schlegel, M. *et al.* Local structure controls the nonaffine shear and bulk moduli of disordered solids. *Sci. Rep.* **6**, 18724; doi: 10.1038/srep18724 (2016).

## Supplementary Material

Supplementary Information

## References

[b1] Born M. & Huang H. Dynamical Theory of Crystal Lattices (Oxford University Press 1954).

[b2] Goodrich C. P., Liu A. J. & Nagel S. R. Solids between the mechanical extremes of order and disorder. Nature Physics (2014).

[b3] Amir A., Krich J., Vitelli V., Oreg Y. & Imry Y. Emergent percolation length and localization in random elastic networks. Phys. Rev. X 3, 021017 (2013).

[b4] Ellenbroek W. G., Zeravcic Z., van Saarloos W. & van Hecke M. Non-affine response: Jammed packings vs. spring networks. EPL 87 34004 (2009).

[b5] Weaire D., Ashby M. F., Logan J. & Weins M. J. On the use of pair potentials to calculate the properties of amorphous metals. Acta Metallurgica 19, 779 (1971).

[b6] Dietsche W., Kinder H., Mattes J. & Wuehl H. Breakdown of Shear Stiffness in Amorphous Ga. Physical Review Letters 45, 1332 (1980).

[b7] Mitzdorf U. & Helmreich D. Elastic constants of *D*_2_*O* ice and variation of intermolecular forces on deuteration. The Journal of Acoustical Society of America 49, 723 (1971).

[b8] Tanaka H., Kawasaki T., Shintani H. & Watanabe K. Critical-like behaviour of glass-forming liquids. Nature Materials 9, 324 (2010). 20173749 10.1038/nmat2634

[b9] Leocmach M., Russo J. & Tanaka H. Importance of many-body correlations in glass transition: An example from polydisperse hard spheres. Journal of Chemical Physics 138, 12A536 (2013). 10.1063/1.4769981 23556787

[b10] Alexander S. Amorphous solids: their structure, lattice dynamics and elasticity. Physics Reports 296, 65–236 (1998).

[b11] Thorpe M. F. Continuous deformations in random networks. J. Non-Cryst. Solids 57, 355–370 (1983).

[b12] Thomson W. (Lord Kelvin), Molecular constitution of matter. Proceedings of the Royal Society of Edinburgh 16, 693–724 (1890).

[b13] Lematre A. & Maloney C. Sum Rules for the Quasi-Static and Visco-Elastic Response of Disordered Solids at Zero Temperature. Journal of Statistical Physics 123, 415–453 (2006).

[b14] Zaccone A. & Scossa-Romano E. Approximate analytical description of the nonaffine response of amorphous solids. Physical Review B 83, 184205 (2011).

[b15] Zaccone A., Blundell J. R. & Terentjev E. M. Network disorder and nonaffine deformations in marginal solids. Physical Review B 84, 174119 (2011).

[b16] Elliott S. R. The Physics and Chemistry of Solids (Wiley, New York, 1998).

[b17] Tilley R. Understanding solids (Wiley, New York, 2013), p. 345.

[b18] Ashcroft N. W. & Mermin N. D. Solid State Physics (Thomson Brooks/Cole, 1976).

[b19] Boolchand P., Lucovsky G., Phillips J. C. & Thorpe M. F. Self-organization and the physics of glassy networks. Phil. Mag. 85, 3823–3838 (2005).

[b20] Clusel M., Corwin E. I., Siemens A. O. N. & Brujic J. A ‘granocentric’ model for random packing of jammed emulsions. Nature 460, 611–615 (2009).

[b21] Rektorys K. Survey of Applicable Mathematics (The M.I.T. Press, Cambridge, Massachusetts, 1969).

[b22] Mason T. G. & Weitz D. A. Elasticity of compressed emulsions. Phys. Rev. Lett. 75, 2051 (1995). 10059196 10.1103/PhysRevLett.75.2051

[b23] Lacasse M. D., Grest G. S., Levine D., Mason T. G. & Weitz D. A. Model for the eleasticity of compressed emulsions. Phys. Rev. Lett. 76, 3448 (1996). 10060969 10.1103/PhysRevLett.76.3448

[b24] Wyart M. In Microgels: Synthesis, Properties, and Applications (Wiley, Weinheim, 2011), p. 95.

[b25] Bechmann R. Elastic and piezoelectric constants of *α*-quartz. Physical Review 110, 1060 (1958).

[b26] Jorjadze I., Pontani L. & Brujic J. Microscopic Approach to the Nonlinear Elasticity of Compressed Emulsions. Physical Review Letters 110, 048302 (2013). 25166208 10.1103/PhysRevLett.110.048302

[b27] Bauer E. & Sigrist M. (Eds), Non-Centrosymmetric Superconductors (Springer, Heidelberg, 2012).

