# Supplementary material for: Local structure controls the nonaffine shear and bulk moduli of disordered solids
Source: Sci Rep. 2016 Jan 6;6:18724. doi: 10.1038/srep18724 (PMC4702120; doi:10.1038/srep18724)
Supplement: Supplementary Information [file srep18724-s1.pdf]

# Supplementary Information for "Local structure controls shear and bulk moduli in disordered solids"

M. Schlegel[1], E. M. Terentjev[1], J. Brujic[2] and A. Zaccone[1,3]
[1] *Cavendish Laboratory, JJ Thomson Avenue, CB30HE Cambridge, U.K.*
[2] *Center for Soft Matter Research, Physics Department, New York University, 4 Washington Place, New York, New York 10003, USA and*
[3] *Physics Department and Institute for Advanced Study, Technische Universität München, 85748 Garching, Germany*

PACS numbers:

## THEORY OF NONAFFINE ELASTIC MODULI IN DISORDERED SOLIDS

We shall give an overview of the nonaffine response formalism and of its mean-field closed-form predictions for the elastic moduli of amorphous solids. The final result of this appendix will be general expressions for the elastic constants accounting for nonaffine displacements due to structural disorder covering the limits of random networks and packings. The difference between the two limits resides in the short-range excluded-volume correlations which are absent in the random networks (where the nodes are point atoms with zero volume), while they play an important role in packings.

According to the nonaffine response formalism developed by various authors in the past, the elastic constants $C_{\iota\xi\kappa\chi}$ for an amorphous solid are given as follows:

$$C_{\iota\xi\kappa\chi} = C^{A}_{\iota\xi\kappa\chi} - C^{NA}_{\iota\xi\kappa\chi}. \tag{1}$$

Here the Cartesian components of the strains are represented by Greek indexes $\iota\xi\kappa\chi$. $C^{A}_{\iota\xi\kappa\chi}$ indicates the affine part which is the Born-Huang term [1]. The nonaffine correction term is represented by $C^{NA}_{\iota\xi\kappa\chi}$. In the harmonic approximation, a quadratic interparticle potential between any two nearest-neighbour particles $i$ and $j$ ($i \neq j$) is assumed, $U_{ij} = \frac{1}{2}\kappa\left(R_{ij} - R_0\right)^2$. Here $\kappa$ represents the bond stiffness and $R_{ij} = \left|\underline{R}_i - \underline{R}_j\right|$ ($\underline{R}_i$ and $\underline{R}_j$ being the position vector of particle $i$ and $j$, respectively) is the equilibrium distance, or bond length, between the two bonded particles. In the following, we shall use $R_0$ for the equilibrium distance. In the harmonic approximation, the affine elastic constants were derived in the following form by Born and his collaborators [1]:

$$C^{A}_{\iota\xi\kappa\chi} = \frac{R_0^2\kappa}{2V}\sum_{ij} c_{ij} n^{\iota}_{ij} n^{\kappa}_{ij} n^{\xi}_{ij} n^{\chi}_{ij} \tag{2}$$

Here $V$ represents the volume of the material, $c_{ij}$ is the occupation matrix ($= 1$ if $i$ and $j$ are bonded, $= 0$ otherwise), $n^{\iota}_{ij}$ is the $\iota$ Cartesian coordinate of the unit vector of the bond. The unit vector in spherical coordinates is given by spherical coordinates:

$$\underline{n}_{ij} = \begin{pmatrix} \sin(\theta_{ij})\cos(\phi_{ij}) \\ \sin(\theta_{ij})\sin(\phi_{ij}) \\ \cos(\theta_{ij}) \end{pmatrix} \tag{3}$$

Here $\theta$ represents the polar angle and $\phi$ the azimuthal angle. In an isotropic disordered solid, the bond unit vector $\underline{n}_{ij}$ can take any value in the solid angle with the same probability $1/4\pi$. Upon introducing the average number of bonds per particle $z$, and upon averaging over the solid angle, one readily obtains the mean-field estimate of the affine elastic constant, e.g. for shear as $G = (1/30)\kappa R_0^2 (N/V) z$.

The non-affine correction is given by [3]:

$$C^{NA}_{\iota\xi\kappa\chi} = \sum_{i<j} \underline{\Xi}^{\iota\xi}_{i} \left(\underline{\underline{H}}_{ij}\right)^{-1} \underline{\Xi}^{\kappa\chi}_{j} \tag{4}$$

where $C^{NA}_{\iota\xi\kappa\chi}$ is a positive non-zero quantity in the presence of structural disorder, hence the non-affinity reduces the elastic energy of the system as can be seen from Eq. (1) [3]. $\underline{\underline{H}}_{ij} = \frac{\partial^2 U_{tot}}{\partial R_i \partial R_j}$ represents the entries of the dynamical (Hessian) matrix $\underline{\underline{H}}$ = of the total interaction energy of all atoms $U_{tot} = \sum_{i<j} U_{ij}$ [3].

We now consider the increment of the local force $\underline{f}_i$ acting on a particle due to the affine displacements of the neighbouring particles. This increment is described by the vector $\underline{\Xi}^{\iota\xi}_{i}$. This force is non-zero only for lattices that lack central symmetry, whereas it is zero for centrosymmetric lattices where each force transmitted by a bonded neighbour vanishes by cancellation with the opposite force transmitted by its mirror-image. Hence, there is a link between local central symmetry and local mechanical equilibrium. For disordered lattices with no central symmetry, mechanical equilibrium is no longer enforced by the lattice symmetry, and the non-vanishing forces need to be relaxed through nonaffine displacements $\underline{f}_i$ In the example of a shear deformation $\gamma$ in the $xy$-plane, the force is given as $\underline{f}_i = \underline{\Xi}^{xy}_{i}\gamma$. As shown in previous works, the following relation holds in the harmonic approximation:

$$\underline{\Xi}^{xy}_{i} = -R_0\kappa\sum_{j} \underline{n}_{ij} n^{x}_{ij} n^{y}_{ij}, \tag{5}$$

where the sum is over the nearest neighbours of the atom $i$. It can be easily verified by insertion that for any simple lattice with central symmetry $\Xi_i^{xy} = 0$.

### The random network limit

Using a mean-field approximation, one can obtain the non-affine part of the elastic constants in closed analytical form, by using general properties of the dynamical matrix and its eigenvectors. If there are no spatial correlations between any two bonds in the solid, as is the case of a random network of point atoms connected by harmonic bonds, then the lattice sums in Eq.(4) has been evaluated in a number of previous studies leading to [4]:

$$C_{\iota\xi\kappa\chi}^{NA} \simeq \kappa R_0^2 3\frac{N}{V}\sum_{\alpha=x,y,z} B_{\alpha,\iota\xi\kappa\chi} \tag{6}$$

where the coefficients $B_{\alpha,\iota\xi\kappa\chi}$ are defined as follows:

$$B_{\alpha,\iota\xi\kappa\chi} = \left\langle n_{ij}^{\alpha} n_{ij}^{\iota} n_{ij}^{\xi} n_{ij}^{\alpha} n_{ij}^{\kappa} n_{ij}^{\chi} \right\rangle. \tag{7}$$

Since $ij$ is not spatially correlated with any other bond in the solid, meaning that its orientation in the solid angle does not depend on the orientation of any other bond, no correlation terms involving bonds other than $ij$ survive the angular averaging. Here

$$\langle ... \rangle = \int_{\theta_{ij}=0}^{\pi} \int_{\phi_{ij}=0}^{2\pi} ... \rho_{ij}\left(\phi_{ij}, \theta_{ij}\right) \sin\left(\theta_{ij}\right) \mathrm{d}\phi_{ij} \mathrm{d}\theta_{ij} \tag{8}$$

represents the orientation average as in [4]. For a disordered isotropic solid, the probability density distribution of bond orientations for a single bond is $\rho_{ij}(\phi_{ij}, \theta_{ij}) = 1/4\pi$, leading to $\sum_{\alpha=x,y,z} B_{xyxy} = 1/15$. Upon replacing this result in the above expression for $C_{\iota\xi\kappa\chi}^{NA}$, the nonaffine shear modulus can be evaluated in closed-form for random networks as $G = (1/30)\kappa R_0^2 (N/V)(z - z_{iso})$, with $z_{iso} = 6$.

### The jammed packing limit

In jammed packings two distinct bonds $ij$ and $lm$ that are far apart, may still be uncorrelated, in the sense that the orientation of $ij$ does not depend on the orientation of $lm$ or viceversa. If, however, the two bonds share a particle, such as $ij$ and $iq$, they are no longer uncorrelated due to the local excluded-volume effect which forbids the overlapping of particles $j$ and $q$. The situation is exemplified in Fig. 1d in the main article. Therefore, the orientation of $ij$ must depend on the orientation of $iq$ because particles $j$ and $q$ cannot overlap in the excluded portion of the solid angle around $i$. Excluded-volume thus leads to an additional correlation term in the expression for the nonaffine part of the elastic constants, which now reads as

$$C_{\iota\xi\kappa\chi}^{NA} \simeq \kappa R_0^2 3\frac{N}{V}\sum_{\alpha=x,y,z} \left(A_{\alpha,\iota\xi\kappa\chi} + B_{\alpha,\iota\xi\kappa\chi}\right) \tag{9}$$

The correlation term has been derived in Ref.[2] and is given by

$$A_{\alpha,\iota\xi\kappa\chi} = \left\langle n_{ij}^{\alpha} n_{ij}^{\iota} n_{ij}^{\xi} n_{iq}^{\alpha} n_{iq}^{\kappa} n_{iq}^{\chi} \right\rangle \tag{10a}$$

$$= \int_{\Omega}\int_{\Omega-\Omega_{cone}} \rho_{ij}(\Omega_{ij} \mid \Omega_{iq}) \rho_{iq}\left(\Omega_{iq}\right) n_{iq}^{\alpha} n_{iq}^{\kappa} n_{iq}^{\chi} \times n_{ij}^{\alpha} n_{ij}^{\iota} n_{ij}^{\xi} \mathrm{d}\Omega_{ij} \mathrm{d}\Omega_{iq}. \tag{10b}$$

Here we use $\rho_{ij}(\Omega_{ij} \mid \Omega_{iq})$ to denote the conditional probability that $ij$ has orientation $\Omega_{ij}$ given that $iq$ has orientation $\Omega_{iq}$. $\Omega$ denotes the total solid angle centered on the spherical particle $i$, while $\Omega_{iq}$ denotes an orientation in the solid angle determined by the pair of angles $(\theta_{iq}, \phi_{iq})$, and, in the same way, $\Omega_{ij}$ is defined by the pair $(\theta_{iq}, \phi_{iq})$. To evaluate the above integral it is necessary to first identify the correlation between $ij$ and $iq$ and then devise a strategy to evaluate the integral in the above equation.

## EVALUATION OF ELASTIC CONSTANTS OF JAMMED PACKINGS

### Excluded-volume cone

Due to excluded-volume correlations, if $\rho_{iq}(\phi_{iq}, \theta_{iq}) = 1/4\pi$, then the orientation of $ij$ must depend on the orientation of $iq$. In other words the probability $\rho_{iq}$ is a free probability, while $\rho_{ij}$ is a conditional probability. The angular space available to $ij$, once the orientation of $iq$ has been fixed, is restricted to $\Omega - \Omega_{cone}$, where $\Omega_{cone}$ denotes the excluded-volume cone centred on the bond $iq$, cfr. Fig.1d in the main article. Hence, the conditional probability density for $ij$ can be written as

$$\rho_{ij}(\Omega_{ij} \mid \Omega_{iq}) = \frac{1}{4\pi - \Omega_{cone}} = \frac{1}{4\pi - \pi\left(\frac{\sigma}{R_0}\right)^2}. \tag{11}$$

In the above expression the excluded-volume cone follows from its definition in terms of the angle $\psi$ which defines the quarter of the aperture of the cone,

$$\psi = \arcsin\left(\frac{\sigma}{2R}\right). \tag{12}$$

The solid angle sector which defines the excluded cone is therefore given by

$$\Omega_{cone} = \int_{\theta=0}^{2\psi}\int_{\phi=0}^{2\pi} \sin(\theta)\, \mathrm{d}\theta \mathrm{d}\phi \tag{13a}$$

$$= \pi\left(\frac{\sigma}{R_0}\right)^2. \tag{13b}$$

For a jammed packing where the particles are just in touch the diameter $\sigma$ is approximately equal to the equilibrium distance $R_0$, and $\rho_{ij}(\Omega_{ij} \mid \Omega_{iq}) \simeq 1/3\pi$.

### Rotation matrix formalism

Next, we consider the following trick to simplify the integral in Eq. 10b by reducing it to a solvable integral with well-defined integration limits in the solid angle. Exploiting the rotational invariance of the system, we change the coordinate frame for the integration over the solid angle in Eq.10b, to a new frame where the polar ($z$-)axis unit vector $\underline{e}_z$ coincides with the unit vector $\underline{n}_{iq}$. This of course amounts to rotating the coordinate frame into a new frame where the parametrization of the allowed (not excluded) portion of solid angle is much simpler, and one only has to exclude the polar angle from 0 to $2\psi$.

The rotation is defined around an axis $\underline{t}$, parallel to $\underline{e}_y$, and perpendicular to both $\underline{e}_z$ and $\underline{n}_{iq}$, with an angle of $\theta_{iq}$ (usual convention of rotation: counter clockwise if axis vector points in the direction of the viewer). Therefore, the unit vector $\underline{t}$ defining the rotation axis is:

$$\underline{t} = \frac{\underline{e}_z \times \underline{n}_{iq}}{|\underline{e}_z \times \underline{n}_{iq}|}. \tag{14}$$

Here $\times$ indicates the cross product. Using this equation, the unit vector $\underline{t}$ defining the rotation axis can be calculated:

$$\underline{t} = \begin{pmatrix} -\sin(\phi_{iq}) \\ \cos(\phi_{iq}) \\ 0 \end{pmatrix}. \tag{15}$$

Fig.1(e) in the main article depicts this rotation for the example $\phi_{iq} = 0$ and $\phi_{ij} = 0$. It can be seen from the figure that the vector $\underline{n}_{ij}$, i.e. the $ij$-bond unit vector in the non-rotated frame, is given through the $ij$-bond unit vector in the rotated frame $\underline{n}_{ij,rot}$ via:

$$\underline{n}_{ij} = \underline{\underline{R}} \cdot \underline{n}_{ij,rot}. \tag{16}$$

The rotation matrix $\underline{\underline{R}}$ is defined by the Rodrigues' formula [5] from which the matrix form is easily derived by comparison).

$$\underline{\underline{R}} = \cos(\theta_{iq})\, \underline{\underline{1}} + \sin(\theta_{iq})\, \left[\underline{t}\right]_\times + (1-\cos(\theta_{iq}))\, \underline{t}\otimes\underline{t} \tag{17}$$

$\underline{\underline{1}}$ represents the identity matrix. Additionally, the following definitions are used, where $t_x$,$t_y$ and $t_z$ represent the components of the $x$-axis, $y$-axis and $z$-axis respectively:

$$\underline{t}\otimes\underline{t} = \begin{pmatrix} t_x^2 & t_xt_y & t_xt_z \\ t_xt_y & t_y^2 & t_yt_z \\ t_xt_z & t_yt_z & t_z^2 \end{pmatrix} = \begin{pmatrix} \sin^2(\phi_{iq}) & -\sin(\phi_{iq})\cos(\phi_{iq}) & 0 \\ -\sin(\phi_{iq})\cos(\phi_{iq}) & \cos^2(\phi_{iq}) & 0 \\ 0 & 0 & 0 \end{pmatrix} \tag{18}$$

$$\left[\underline{t}\right]_\times = \begin{pmatrix} 0 & -t_z & t_y \\ t_z & 0 & -t_x \\ -t_y & t_x & 0 \end{pmatrix} = \begin{pmatrix} 0 & 0 & \cos(\phi_{iq}) \\ 0 & 0 & \sin(\phi_{iq}) \\ -\cos(\phi_{iq}) & -\sin(\phi_{iq}) & 0 \end{pmatrix}. \tag{19}$$

Next, we look at the integral $I$ defined as:

$$I_{\alpha\iota\xi} = \int_{\Omega-\Omega_{\mathrm{cone}}} n_{ij}^{\alpha} n_{ij}^{\iota} n_{ij}^{\xi} \sin(\theta_{ij})\, \mathrm{d}\theta_{ij}\mathrm{d}\phi_{ij}. \tag{20}$$

This integral occurs in the expression for $A_{\alpha,\iota\xi\kappa\chi}$, and considering that $\rho_{ij}(\Omega_{ij} \mid \Omega_{iq}) = const$ in the allowed solid angle $\Omega - \Omega_{\mathrm{cone}}$ for $ij$, we have factored $\rho_{ij}(\Omega_{ij} \mid \Omega_{iq}) = const$ out of the $ij$ integral leaving a product between $I_{\alpha\iota\xi}$ and $\rho_{ij}(\Omega_{ij} \mid \Omega_{iq})$ inside the integral for $A_{\alpha,\iota\xi\kappa\chi}$,

$$A_{\alpha,\iota\xi\kappa\chi} = \int_{\Omega}\int_{\Omega-\Omega_{\mathrm{cone}}} \rho_{ij}(\Omega_{ij} \mid \Omega_{iq})\rho_{iq}(\Omega_{iq})\, n_{iq}^{\alpha} n_{iq}^{\kappa} n_{iq}^{\chi} \times n_{ij}^{\alpha} n_{ij}^{\iota} n_{ij}^{\xi} \mathrm{d}\Omega_{ij}\mathrm{d}\Omega_{iq} \tag{21a}$$

$$= \int_{\Omega} I_{\alpha\iota\xi}\rho_{ij}(\Omega_{ij} \mid \Omega_{iq})\rho_{iq}(\Omega_{iq})\, n_{iq}^{\alpha} n_{iq}^{\kappa} n_{iq}^{\chi} \mathrm{d}\Omega_{iq}. \tag{21b}$$

As next step, one can rewrite the $I_{\alpha\iota\xi}$ integral by transforming from spherical coordinates to Cartesian coordinates:

$$I_{\alpha\iota\xi} = \int_{\Omega-\Omega_{cone}} n_{ij}^{\alpha} n_{ij}^{\iota} n_{ij}^{\xi} \sin(\theta_{ij})\, \mathrm{d}\theta_{ij}\mathrm{d}\phi_{ij} \tag{22a}$$

$$= \int_{\Omega-\Omega_{cone}}\int_{r_{ij}=0}^{\infty} n_{ij}^{\alpha} n_{ij}^{\iota} n_{ij}^{\xi} \delta(r_{ij}-R_0) \tag{22b}$$

$$\times \sin(\theta_{ij})\, \mathrm{d}\theta_{ij}\mathrm{d}\phi_{ij}\mathrm{d}r_{ij} \tag{22c}$$

$$= \int_{V-V_{cone}} n_{ij}^{\alpha} n_{ij}^{\iota} n_{ij}^{\xi} \delta(r_{ij}-R_0)\, \mathrm{d}x_{ij}\mathrm{d}y_{ij}\mathrm{d}z_{ij}. \tag{22d}$$

We now define the transformation of rotated coordinates $\underline{r}_{ij,rot}$ to non-rotated coordinates $\underline{r}_{ij}$ as:

$$\underline{r}_{ij} = \Phi\left(\underline{r}_{ij,rot}\right) \equiv \underline{\underline{R}} \cdot \underline{r}_{ij,rot}, \tag{23}$$

where $\underline{\underline{R}}$ is the rotation matrix defined in Eq. (17). In the following D$\Phi$ denotes the Jacobian matrix of the function $\Phi$ and det (D$\Phi$) is the determinant of the Jacobian. Using the transformation theorem in three dimensions [6], one obtains:

$$I = \int_{[V-V_{cone}]_{rot}} n_{ij}^{\alpha} n_{ij}^{\iota} n_{ij}^{\xi} \delta\left(r_{ij,rot} - R_0\right) \times \left|\det\left(\mathrm{D}\Phi\right)\right| \mathrm{d}x_{ij,rot} \mathrm{d}y_{ij,rot} \mathrm{d}z_{ij,rot} \tag{24a}$$

$$= \int_{[V-V_{cone}]_{rot}} n_{ij}^{\alpha} n_{ij}^{\iota} n_{ij}^{\xi} \delta\left(r_{ij,rot} - R_0\right) \times \mathrm{d}x_{ij,rot} \mathrm{d}y_{ij,rot} \mathrm{d}z_{ij,rot} \tag{24b}$$

$$= \int_{[\Omega-\Omega_{cone}]_{rot}} \int_{\tilde{r}_{ij}=0}^{\infty} n_{ij}^{\alpha} n_{ij}^{\iota} n_{ij}^{\xi} \delta\left(\tilde{r}_{ij} - R_0\right) \times \sin\left(\tilde{\theta}_{ij}\right) \mathrm{d}\tilde{\theta}_{ij} \mathrm{d}\tilde{\phi}_{ij} \mathrm{d}\tilde{r}_{ij} \tag{24c}$$

$$= \int_{[\Omega-\Omega_{cone}]_{rot}} n_{ij}^{\alpha} n_{ij}^{\iota} n_{ij}^{\xi} \sin\left(\tilde{\theta}_{ij}\right) \mathrm{d}\tilde{\theta}_{ij} \mathrm{d}\tilde{\phi}_{ij} \tag{24d}$$

$$= \int_{\tilde{\theta}_{ij}=\theta_{min}}^{\pi} \int_{\tilde{\phi}_{ij}=0}^{2\pi} n_{ij}^{\alpha} n_{ij}^{\iota} n_{ij}^{\xi} \sin\left(\tilde{\theta}_{ij}\right) \mathrm{d}\tilde{\theta}_{ij} \mathrm{d}\tilde{\phi}_{ij}. \tag{24e}$$

Here we used $\mathrm{D}\Phi = \underline{\underline{R}}$ and $\left|\det\left(\underline{\underline{R}}\right)\right| = 1$ for rotation matrices $\underline{\underline{R}}$ [6]. $\theta_{min}$ is determined by the excluded volume cone [2] as:

$$\theta_{min} = 2\psi = 2 \cdot \arcsin\left(\frac{\sigma}{2R_0}\right). \tag{25}$$

We recall that $n_{ij}^{\alpha}$ is defined as the $\alpha$ Cartesian coordinate of the bond unit vector $\underline{n}_{ij}$ and is related to the bond unit vector of the rotated frame $\underline{n}_{ij,rot}$ via $\underline{n}_{ij} = \underline{\underline{R}} \cdot \underline{n}_{ij,rot}$. In the rotated frame, $\underline{n}_{ij,rot}$ is given by:

$$\underline{n}_{ij,rot} = \begin{pmatrix} \sin\left(\tilde{\theta}_{ij}\right) \cos\left(\tilde{\phi}_{ij}\right) \\ \sin\left(\tilde{\theta}_{ij}\right) \sin\left(\tilde{\phi}_{ij}\right) \\ \cos\left(\tilde{\theta}_{ij}\right) \end{pmatrix}. \tag{26}$$

Therefore, we can now use Eq.(25e) together with Eq.(21b) to arrive at the following expression for $A_{\alpha,\iota\xi\kappa\chi}$:

$$A_{\alpha,\iota\xi\kappa\chi} = \left\langle n_{ij}^{\alpha} n_{ij}^{\iota} n_{ij}^{\xi} n_{iq}^{\alpha} n_{iq}^{\kappa} n_{iq}^{\chi} \right\rangle \tag{27a}$$

$$= \int_{\Omega} \int_{\Omega-\Omega_{cone}} \rho_{ij} \rho_{iq} n_{iq}^{\alpha} n_{iq}^{\kappa} n_{iq}^{\chi} n_{ij}^{\alpha} n_{ij}^{\iota} n_{ij}^{\xi} \mathrm{d}\Omega_{ij} \mathrm{d}\Omega_{iq} \tag{27b}$$

$$= \int_{\theta_{iq}=0}^{\pi} \int_{\phi_{iq}=0}^{2\pi} \int_{\tilde{\theta}_{ij}=2\psi}^{\pi} \int_{\tilde{\phi}_{ij}=0}^{2\pi} \rho_{ij} \rho_{iq} n_{iq}^{\alpha} n_{iq}^{\kappa} n_{iq}^{\chi} \, . \times n_{ij}^{\alpha} n_{ij}^{\iota} n_{ij}^{\xi} \sin\left(\tilde{\theta}_{ij}\right) \sin\left(\theta_{iq}\right) \mathrm{d}\tilde{\theta}_{ij} \mathrm{d}\tilde{\phi}_{ij} \mathrm{d}\theta_{iq} \mathrm{d}\phi_{iq} \tag{27c}$$

With the last identity Eq.(27c), we have reduced the original integral for $A_{\alpha,\iota\xi\kappa\chi}$ to a much simpler integral with well-defined integration limits in the solid angle.

## Evaluation of excluded-volume correlation terms in the elastic moduli

In fact, we arrived at an expression for $A_{\alpha,\iota\xi\kappa\chi}$ as an integral of two independent sets of variables $\{\tilde{\theta}_{ij}, \tilde{\phi}_{ij}\}$ and $\{\theta_{iq}, \phi_{iq}\}$. The integration can be easily done numerically or analytically and leads to the following values for the excluded volume coefficients $A_{\alpha,\iota\xi\kappa\chi}$,

| $\alpha$ | $x$ | $y$ | $z$ |
|---|---|---|---|
| $A_{\alpha,xxxx}$ | −0.0304 | −0.00357 | −0.00357 |
| $A_{\alpha,xyxy}$ | −0.00357 | −0.00357 | −0.000149 |
| $A_{\alpha,xxyy}$ | −0.00982 | −0.00982 | −0.00327 |

(28)

These coefficients can be used in Eq.(9) to determine the nonaffine contributions to the elastic constants.

The orientation-averaged affine contribution is $C_{\iota\xi\kappa\chi}^{A} = \kappa R_0^2 \frac{zN}{2V} \langle n_{ij}^{\iota} n_{ij}^{\kappa} n_{ij}^{\xi} n_{ij}^{\chi} \rangle$, where $\langle ... \rangle = \int_0^{\pi} \int_0^{2\pi} \frac{1}{4\pi} ... d\phi_{ij} \sin\theta_{ij} d\theta_{ij}$, since the bond $ij$ can have any orientation in the solid angle with the same likelihood $1/4\pi$. We also recall that $B_{x,xxxx} = 1/7$, $B_{y,xxxx} = 1/35$, $B_{z,xxxx} = 1/35$ $B_{x,xyxy} = B_{y,xyxy} = 1/35$, $B_{x,xxyy} = B_{y,xxyy} = 1/35$, $B_{z,xyxy} = B_{z,xxyy} 1/105$ as obtained in Ref. [4]. It is important to notice that the affine contribution to the moduli is proportional to the coordination number $z$, because the storage elastic energy is proportional to the total number of bonds ($zN/2$) in the solid. With these values of the coefficients $A_{\alpha,\iota\xi\kappa\chi}$ and $B_{\alpha,\iota\xi\kappa\chi}$ in Eq. (9), and then in Eq.(1) it is possible to evaluate the elastic moduli $G$ and $K$ accounting for short-range excluded-volume correlations, which are plotted in Fig. (2a) and (2b) in the main article.


[1] Born, M. and Huang, H., Dynamical Theory of Crystal Lattices, (Oxford University Press 1954).

[2] Zaccone, A. and Terentjev, E. M. Short-range correlations control the G/K and Poisson ratios of amorphous solids and metallic glasses. *Journal of Applied Physics* **115,** 033510 (2014).

[3] Lemaître, A. and Maloney, C., Sum Rules for the Quasi-Static and Visco-Elastic Response of Disordered Solids at Zero Temperature. *Journal of Statistical Physics* **123,** 415-453 (2006).

[4] Zaccone, A. & Scossa-Romano, E., Approximate analytical description of the nonaffine response of amorphous solids. *Physical Review B* **83,** 184205 (2011).

[5] Morse and Feshbach, Mathematical methods of theoretical physics.

[6] Rektorys, K., *Survey of Applied Mathematics* (The M.I.T. Press, Cambridge, Massachusetts, 1969).
